# Supplementary material for: HAPI: An efficient Hybrid Feature Engineering-based Approach for Propaganda Identification in social media
Source: PLoS One. 2024 Jul 10;19(7):e0302583. doi: 10.1371/journal.pone.0302583 (PMC11236156; doi:10.1371/journal.pone.0302583)
Supplement: Attachment — Submitted filename: Response to Reviewers (27.03.2024).pdf [file pone.0302583.s001.pdf]

Original Manuscript ID:

Original Article Title: "HAPI: An Efficient Hybrid Feature Engineering-based Framework for Propaganda Identification in Social Media"

Updated Article Title: "HAPI: An Efficient Hybrid Feature Engineering-based Approach for Propaganda Identification in Social Media"

To: Plos ONE Editor

Re: Response to reviewers

Dear Editor,

Thank you for allowing a resubmission of our manuscript, with an opportunity to address the reviewers' comments.

We are uploading (a) our point-by-point response to the comments (below) (response to reviewers, under "Author's Response Files*"*), (b) an updated manuscript with yellow highlighting indicating changes (as "Highlighted PDF*"*), and (c) a clean updated manuscript without highlights ("Main Manuscript"*).*

Thank you for your dedication throughout the review process.

Best regards,

<Mudasir Wani> et al

Editorial, Requirement # 1 Please ensure that your manuscript meets PLOS ONE's style requirements, including those for file naming.

Author response: We followed the PLOS ONE's latex template for the style requirements and all the files are renamed as mentioned in the guidelines.

Editorial, Requirement # 2 (1) In your Methods section, please include additional information about your dataset and ensure that you have included a statement specifying whether the collection and analysis method complied with the terms and conditions for the source of the data.

Author Actions:

We have now included the dataset statistics and data collection process in detail including whether collection and analysis methods obey the terms and conditions specified by the data source in the revised manuscript.

The newly added content is highlighted in the revised manuscript.

---

(2) Please note that PLOS ONE has specific guidelines on code sharing for submissions in which author-generated code underpins the findings in the manuscript. In these cases, all author-generated code must be made available without restrictions upon publication of the work

Author Actions:

In compliance with the code-sharing policy of PLOS ONE, we ensured that all author-generated code used to support the findings presented in our manuscript is made openly accessible on our GitHub account. The link to the repository is provided in the Data availability statement. This will include providing access to the code through a publicly accessible git repository. We have taken the necessary steps to prepare the code for sharing and have included details on how to access it in the revised manuscript.

Editor, Requirement# 3 : In this instance, it seems there may be acceptable restrictions in place that prevent the public sharing of your minimal data. However, in line with our goal of ensuring long-term data availability to all interested researchers, PLOS' Data Policy states that authors cannot be the sole named individuals responsible for ensuring data access (http://journals.plos.org/plosone/s/data-availability#loc-acceptable-data-sharing-methods).

Data requests to a non-author institutional point of contact, such as a data access or ethics committee, helps guarantee long term stability and availability of data. Providing interested researchers with a durable point of contact ensures data will be accessible even if an author changes email addresses, institutions, or becomes unavailable to answer requests.

Before we proceed with your manuscript, please also provide non-author contact information (phone/email/hyperlink) for a data access committee, ethics committee, or other institutional body to which data requests may be sent. If no institutional body is available to respond to requests for your minimal data, please consider if there any institutional representatives who did not collaborate in the study, and are not listed as authors on the manuscript, who would be able to hold the data and respond to external requests for data access? If so, please provide their contact information (i.e., email address). Please also provide details on how you will ensure persistent or long-term data storage and availability.

Authors' Response: We sincerely appreciate your thorough review of our manuscript and your insightful comments regarding data accessibility by PLOS' Data Policy.

In response to your valuable feedback, we have taken the necessary steps to designate a non-author contact who possesses the requisite administrative access to our dataset. She has extensive experience and expertise in the field and is well-suited to handle data access requests from fellow researchers and ensure compliance with data-sharing policies.

You may find Dr. Shakil's contact information below for your reference:

Name: Dr. Kashish Ara Shakil
Designation: Associate Professor
Department: College of Computer and Information Sciences
Institution: Princess Nora bint Abdulrahman University
Country: Riyadh, KSA
Phone: +966 0545009560
Email: kashakil@pnu.edu.sa

We assure you that Dr. Shakil is fully committed to upholding the principles of data accessibility and will diligently oversee the process of responding to requests, thereby ensuring the persistent and long-term availability of our dataset.

Editor, Requirement# 4: We note that Figures 6 and 7 in your submission contain copyrighted images. All PLOS content is published under the Creative Commons Attribution License (CC BY 4.0), which means that the manuscript, images, and Supporting Information files will be freely available online, and any third party is permitted to access, download, copy, distribute, and use these materials in any way, even commercially, with proper attribution.

Author response: We sincerely appreciate your thorough review of our manuscript, as per the suggestion we have updated the word clouds with different colors and shape.

Author action: We updated the manuscript by modifying the Figure 6 and 7 with different shapes and colors. Although the previous version had no copyright issues as we draw that using python library, in the modified version we changed the shape and colors in order to be it more visible.

Reviewer#1, Concern # 1 (Check acronyms are written in full the first time e.g., OSNs):

Author response: Thank you for assessing this, we have checked all the acronyms and have modified the manuscript by adding their full forms in their first appearance.

Author action: We updated the manuscript by providing the full form of acronyms when mentioned in the first appearance.

Reviewer#1, Concern # 2 (Check Size of some Visuals e.g. figure 2, figure 5 and figure 13):

Author response: We appreciate your assessment that figures 2, 5, and 13 are modified.

Author action: We updated the manuscript by modifying the figures 2,5 and 13.

Reviewer#1, Concern # 3 (Check word cloud construction (Figure 5 and 7) can these be enlarged or their colour change not all words were visible)

Author response: Thank you for pointing this out, as per the suggestion we have updated the word clouds with different colors and shapes.

Author action: We updated the manuscript by modifying Figures 6 and 7 with different shapes and colors. Although the previous version had no copyright issues as we drew that using the Python library, in the modified version we changed the shape and colors in order to make it more visible.

Reviewer#1, Concern # 4 (There is some repetition in the conclusion from the abstract and paper itself, the conclusion could be improved e.g. potential use cases)

Author response: Thank you for your valuable feedback. We acknowledge the repetition in the conclusion and agree that enhancing the conclusion with potential use cases would improve the overall clarity and impact of the paper.

Author Action: In response to the reviewer's feedback, we have revised the manuscript by eliminating repetitions in both the conclusion and abstract sections. Additionally, we have included new content to enhance the details provided in these sections. The new updated and newly added content is highlighted in the revised manuscript.

We appreciate the valuable suggestion and have taken steps to address it in the revised manuscript.

Reviewer#2, Concern # 1 (The title "HAPI: An efficient Hybrid Feature Engineering-based Framework for Propaganda Identification in Social Media" lacks clarity regarding the meaning of 'A' in the abbreviation. I recommend that the authors revise the title accordingly for clarity.)

Author response: Thank you for pointing this out, we have modified the title to "HAPI: An efficient **H**ybrid Feature Engineering-based **A**pproach for **P**ropaganda **I**dentification in Social Media."

Author action: We updated the manuscript by modifying the title to "HAPI: An efficient Hybrid Feature Engineering-based Approach for Propaganda Identification in Social Media" such that the meaning of 'A' in the abbreviation is clear.

Reviewer#2, Concern # 2 (It is advisable to define and elaborate on all abbreviations and acronyms upon their first appearance in the paper.)

Author response: We appreciate your assessment and we have checked all the acronyms and elaborated on their first appearance.

Author action: We updated the manuscript by elaborating all the acronyms in their first appearance.

Reviewer#2, Concern # 3 (The abstract appears rushed and lacks sufficient detail, particularly regarding comparisons and future research directions. Enhancing these aspects would improve the quality of the abstract.)

Author response: Thank you for your feedback on the abstract. We appreciate your insights regarding the need for more detail, particularly in terms of comparisons and future research directions.

Author action: As per the reviewer's suggestions we have now revised the abstract to provide a more comprehensive overview of the study, including clearer comparisons with existing research and a discussion of potential future research directions.

Reviewer#2, Concern # 4 (The literature review section would benefit from a comprehensive concluding paragraph that summarizes the key points drawn from the literature in a clear manner.)

Author response: We appreciate your assessment and we have added the concluding paragraph that summarizes the key findings from the literature.

Author action: We updated the manuscript by adding a concluding paragraph "News article analysis has been the main focus of most research in this area. On the other hand people in the modern era share their thoughts and viewpoints on social media platforms, and there is a great potential to use online social media data for propaganda detection. These platforms are excellent resources for current events and provide insightful information about the propagation of propaganda and its impact on public opinion. The performance of several machine learning classifiers in this context has not been sufficiently enhanced by feature selection techniques. By determining the most relevant and discriminative features in the dataset, feature selection will play a critical role in managing the effectiveness of classifiers."

Reviewer#2, Concern # 5 (It is recommended to provide a definition of propaganda and its synonyms at the beginning of the introduction to facilitate reader understanding.)

Author response: Thank you for pointing this out, we have added the definition of propaganda in the beginning of the introduction.

Author action: We updated the manuscript by adding a definition of propaganda at start of the introduction "Propaganda refers to the dissemination of biased or misleading information in order to influence the opinions, beliefs, attitudes, or behaviors of individuals or groups in a particular direction"

Reviewer#2, Concern # 6 (The authors are required to furnish a detailed elucidation of the word clouds depicted in figures 6 and 7. Furthermore, if feasible, they should offer exemplar sentences corresponding to select words within both categories derived from the amassed dataset.)

Author response: Thank you for pointing this out, as per the suggestion we have updated the word clouds with different colors and shapes.

Author action: We updated the manuscript by modifying Figures 6 and 7 with different shapes and colors. Although the previous version had no copyright issues as we drew that using the python library, in the modified version we changed the shape and colors in order to make it more visible. Also, the words that are more frequent are mentioned in the revised manuscript "Upon close examination, it is evident that posts with propagandistic content tend to revolve on particular subjects, such as politics, religion, trending events, and influential people. This finding is corroborated by a detailed examination of the word cloud linked to propaganda, which shows that terms pertaining to politics, religion, ISIS, and Islam appear most frequently. This implies a deliberate attempt to advance specific ideologies or agendas by disseminating inaccurate or biased information on a variety of digital platforms.".

Reviewer#2, Concern # 7 (Figure 2, depicting the flowchart of processes, suffers from poor visibility, and the caption requires clarification. The authors are urged to improve the figure's quality and update the caption accordingly in the revised version.)

Author response: We have modified Figure 2 and updated its caption for better clarification.

Author action: We updated the revised manuscript by modifying Figure 2.

Reviewer#2, Concern # 8 (Figure 3, illustrating the framework for data extraction from Twitter, appears stretched and requires correction.)

Author response: We appreciate your assessment and we have checked Figure 3 and modified its dimensions.

Author action: We updated the manuscript by modifying the size of Figure 3.

Reviewer#2, Concern # 9(A pictorial representation of the Data Preprocessing (section 2.2) would enhance the clarity and understanding of the process.)

Author response: Thanks for the suggestion, as per the suggestion we have added the pictorial representation of the Data Preprocessing section.

Author action: We updated the manuscript by adding figure 8, which depicts the major steps in data preprocessing.

Reviewer#2, Concern # 10 (The title of section 3, "Results of Propaganda Identification based on HaPi," lacks clarity and should be revised for better coherence.)

Author response: We appreciate your assessment and we have modified the title of section 3.

Author action: We updated the manuscript by changing the title of section 3 to "Results and Discussion".

Reviewer#2, Concern # 11 (Algorithm 1, detailing the HAPI approach, appears improperly formatted. The authors are encouraged to present it in a well-defined format suitable for journal standards.)

Author response: We have rectified the formatting issues in the Algorithm 1.

Author action: We updated the manuscript by modifying the Algorithm 1.

Reviewer#2, Concern # 12 (Several section numbers are incorrect throughout the manuscript and should be updated accordingly.)

Author response: We appreciate your assessment and we have checked the section numbering and rectified the errors in numbering.

Author action: We updated the manuscript by following the numbering style in each section.

Reviewer#2, Concern # 13 (Figure 12, depicting the Comparison of Machine Learning Classifiers based on HAPI, suffers from blurriness, and requires enhancement for improved clarity. Overall, while the paper presents valuable insights into propaganda identification in social media, addressing the concerns and incorporating the suggested improvements would significantly enhance its quality and readability.)

Author response: Thank you for your valuable feedback. We acknowledge the concern regarding the clarity of Figure 12 and recognize the importance of enhancing its quality for improved readability. We have modified Figure 12 and its resolution.

Author action: We updated the manuscript by modifying Figure 12 to look more clear, readable and visible.

Reviewer#2, Concern # 14 (I further suggest removing references to the arxiv papers or replace them with their peer-reviewed versions.)

Author response: We appreciate your assessment and we have checked the arxiv papers and replaced them with their peer review, if available.

Author action: We updated the manuscript by modifying the references of arxiv papers with their peer-reviewed versions.

All the authors once again would like to extend sincere thanks to the editors and reviewers for their valuable feedback on the manuscript. Your input has greatly improved the quality of our work. Thank you for your dedication throughout the review process.
